# Genotypic Characterization of Virulence Factors in Extended-Spectrum Beta-Lactamase (ESBL)-Producing *Escherichia coli* Strains from Chickens in Hungary

**DOI:** 10.3390/antibiotics14111083

**Published:** 2025-10-27

**Authors:** Ádám Kerek, Ábel Szabó, Gergely Tornyos, Eszter Kaszab, Krisztina Bali, Ákos Jerzsele

**Affiliations:** 1 Department of Pharmacology and Toxicology, University of Veterinary Medicine Budapest, H-1078 Budapest, Hungary; 2 National Laboratory of Infectious Animal Diseases, Antimicrobial Resistance, Veterinary Public Health and Food Chain Safety, University of Veterinary Medicine Budapest, H-1078 Budapest, Hungary; 3 One Health Institute, University of Debrecen, Nagyerdei Krt. 98, H-4032 Debrecen, Hungary; 4 Department of Microbiology and Infectious Diseases, University of Veterinary Medicine, István u 2, H-1078 Budapest, Hungary

**Keywords:** *Escherichia coli*, virulence factors, NGS, ExPEC, UPEC, APEC, NMEC

## Abstract

**Background**: The increasing attention on extended-spectrum beta-lactamase (ESBL)-producing *Escherichia coli* strains isolated from poultry flocks stems from concerns about their virulence potential and zoonotic risk. Of particular significance is the identification of extraintestinal pathogenic *E. coli* (ExPEC) pathotypes in poultry, as these strains pose not only animal health concerns but also serious threats to food safety and public health. Mapping the genetic background of pathogenicity and antimicrobial resistance is essential for risk assessment and the development of effective control strategies. **Methods**: A total of 87 *E. coli* isolates were isolated from tracheal and cloacal swab samples collected from healthy chickens between 2022 and 2023. Whole-genome sequencing was performed using Illumina and MGI next-generation sequencing platforms. Bioinformatic analyses were conducted to identify virulence-associated genes and pathotype markers using multiple reference databases, including VirulenceFinder. The frequency of virulence genes was summarized both in tabular form and visualized through graphical representations. **Results**: A substantial proportion of the isolates harbored virulence genes linked to various ExPEC pathotypes, particularly uropathogenic *E. coli* (UPEC), avian pathogenic *E. coli* (APEC), and neonatal meningitis-causing *E. coli* (NMEC). The most frequently detected colonization factors included members of the *fim*, *pap*, *ecp*, and *fae* gene families. Among fitness-related genes, iron acquisition systems—*ent*, *chu*, *iro*, *iuc*, *fep*, and *ybt*—were especially prevalent. Classic UPEC-associated genes such as *pap* and *fimH*, along with the APEC-related *iutA* and *vat*, were found at high frequencies. Four isolates exhibited a virulence gene profile characteristic of the NMEC pathotype (*ibeA*, *kpsD*/*M*/*T*, *fimH*). In contrast, hallmark genes of enteric pathotypes were absent from all isolates. **Conclusions**: The predominance of extraintestinal virulence factors in the examined poultry-derived *E. coli* strains underscores their zoonotic potential. The complete absence of enteric pathotype markers indicates that the studied poultry populations primarily harbor ExPEC-like strains. These findings highlight the critical need for ongoing genomic surveillance and targeted preventive strategies within poultry production systems.

## 1. Introduction

*Escherichia coli* is a rod-shaped, Gram-negative bacterium belonging to the family Enterobacteriaceae [1]. While commonly present as a commensal in the gastrointestinal tract, even non-pathogenic strains may cause disease in hosts with compromised barriers or weakened immunity [2,3].

Certain *E. coli* strains are capable of causing both intestinal and extraintestinal diseases in humans and animals alike [4]. Pathogenic *E. coli* are classified into pathotypes based on their virulence traits, most of which are encoded by mobile genetic elements (MGEs) such as plasmids and transposons. These elements facilitate horizontal gene transfer, enabling diverse combinations of virulence factors to arise. Once integrated into the bacterial genome, these virulence-associated regions may become stable features of the organism [5].

Pathotypes are broadly grouped based on clinical manifestations. The first group encompasses diarrheagenic *E. coli* (DEC), including six main categories: enteropathogenic (EPEC), enterohemorrhagic (EHEC), enterotoxigenic (ETEC), enteroaggregative (EAEC), enteroinvasive (EIEC), and diffusely adherent *E. coli* (DAEC). The second group includes extraintestinal (ExPEC) strains causing urinary tract infections (primarily uropathogenic *E. coli*, UPEC), while the third group comprises strains associated with neonatal meningitis and sepsis (NMEC). A distinct category is avian pathogenic *E. coli* (APEC), which can lead to respiratory disease, pericarditis, and septicemia in poultry [5,6,7].

Many of these pathotypes are of serious public health concern due to their foodborne transmission potential and history of causing large-scale outbreaks in both developing and developed regions [8,9]. The associated virulence genes encode functions such as adhesion, invasion, iron acquisition, motility, and toxin production. These factors are typically classified into colonization, fitness, toxin, and effector modules [10]. The emergence of novel pathotypes can trigger deadly outbreaks, highlighting the need for effective surveillance systems. Given that food, water, animals, and humans all serve as possible vectors for enteric *E. coli*, broad-based monitoring is essential [8,11].

High-throughput sequencing technologies are increasingly used as a powerful surveillance tool, supporting not only outbreak tracing but also the development of new therapeutic strategies and the design of more effective vaccines [12]. Alongside molecular monitoring, strict adherence to biosecurity protocols [13] and the growing application of antibiotic alternatives—such as plant extracts and essential oils [14,15,16,17,18], antimicrobial peptides [19], probiotics, and other biologics [20,21,22]—play a critical role in reducing antimicrobial use. This, in turn, decreases the selective pressure on microbial populations and helps preserve the long-term efficacy of antibiotics in treating *E. coli* infections within the framework of the One Health concept.

In Hungary, *E. coli* infections are a leading cause of economic losses in poultry production, particularly due to colibacillosis caused by APEC strains. Several reports have documented high isolation rates of APEC strains from broilers and layers, with prevalence estimates ranging from 30% to 70%, depending on farm conditions and biosecurity practices [23,24]. These strains not only impact animal health and productivity but also pose a zoonotic risk via the food chain.

Despite the growing interest in antimicrobial resistance and zoonotic potential of *E. coli*, there is limited published data on the virulence profiles and pathotype distribution of ESBL-producing strains circulating in Hungarian poultry. This study represents the first in-depth analysis of virulence gene repertoires in poultry-derived, ESBL-producing *E. coli* strains from Hungary, focusing on the coexistence of multiple pathotype-associated markers, particularly the overlap between ExPEC and other virulence traits in multidrug-resistant isolates. This study was designed to help address this knowledge gap by characterizing their virulence gene content and pathotype affiliations.

Domestic chickens (*Gallus gallus domesticus*) represent one of the most widely consumed sources of animal protein globally and play a central role in the epidemiology of zoonotic pathogens, including *E. coli*. Their intensive production and close interaction with humans via the food chain make them a critical target for One Health-oriented surveillance efforts [25,26]. Furthermore, poultry farms have been identified as major reservoirs for pathogenic and multidrug-resistant *E. coli* strains, highlighting the need for regular monitoring. While several studies from other European countries (e.g., Poland, Germany, Italy) have reported on virulence traits in poultry-associated *E. coli*, comparative data from Central and Eastern Europe remain sparse [27,28,29]. Moreover, contrasting these findings with recent datasets from Asia may help reveal regional variations in virulence gene distribution and provide a broader epidemiological context.

In this study, we aimed to characterize the virulence gene repertoire of *E. coli* strains isolated from chickens and evaluate their potential pathotypes in light of One Health priorities.

## 2. Results

### 2.1. Identified Virulence Gene Repertoire

A total of 87 *E. coli* isolates isolated from chickens, which had been confirmed as extended-spectrum beta-lactamase (ESBL) producers by both phenotypic and genotypic screening, were subjected to genomic characterization to investigate their virulence gene repertoire. The analysis of colonization-associated virulence factors (Table 1) revealed the presence of thirty-nine distinct genes, of which 26 (66.7%) were associated with UPEC, 11 (28.2%) with ETEC, and 2 (5.1%) with ExPEC-related traits. Among the 87 ESBL-producing *E. coli* isolates, UPEC-associated genes were present in 58 strains (66.7%), ETEC-characteristic genes in 63 strains (72.4%), and ExPEC-related colonization factors in 49 strains (56.3%). Representative UPEC markers such as *fimH*, *papG*, and *yagZ*/*ecpA* were frequently detected, alongside ETEC-associated fimbrial genes including *faeC*–*faeJ* and members of the *csg* family. ExPEC-related colonization factors, such as *fdeC* and *ompA*, were also identified in a considerable proportion of isolates (Supplementary Materials, Additional data (chickens)).

The complete presence of the *fim* and *pap* gene families—particularly the co-occurrence of *fimH*, *papG*, and *ompA*—is strongly indicative of the presence of UPEC-like pathotypes, even in poultry-derived strains, suggesting potential zoonotic relevance. Additionally, the full representation of the *E. coli* common pilus (ECP) operon (*yagV*–*ykgK*) further supports the substantial colonization capacity of these isolates, especially in relation to UPEC and broader ExPEC lineages.

As shown in Table 2, a diverse range of genes related to siderophore systems and heme utilization were identified among the poultry-derived *E. coli* strains. The *chu* gene cluster (*chuA*, *chuS*, *chuT*, *chuU*, *chuV*, *chuW*, *chuX*, *chuY*) was detected in 22 out of 87 isolates (25.3%), suggesting a functional capability for iron acquisition directly from heme. Similarly, the *shu* gene system (*shuA*, *shuS*, *shuT*, *shuX*, *shuY*) was identified in 19 isolates (21.8%), indicating potential redundancy in heme-based iron uptake mechanisms.

Among the siderophore-associated genes, components from multiple systems were identified. The classical enterobactin biosynthetic and transport machinery was nearly fully represented (*entA*–*F*, *entS*, *fepA*–*G*, *fes*), alongside genes associated with the salmochelin (*iroB*–*E*, *iroN*), yersiniabactin (*irp1*, *irp2*, *ybtA*, *ybtE*, *ybtP*–*X*), and aerobactin (*iucA*–*D*, *iutA*) pathways. While many of these genes are generally classified as ExPEC-associated virulence factors, several—particularly *iroN*, *fyuA*, and *iutA*—are pathotype-specific markers commonly linked to APEC and UPEC strains.

Siderophore systems were widely distributed among the isolates. A total of 9 isolates (14.3%) harbored components of all four major siderophore systems (enterobactin, aerobactin, salmochelin, and yersiniabactin), while 7 isolates (11.1%) carried three, 25 isolates (39.7%) carried two, and 19 isolates (30.2%) carried one. This cumulative presence of multiple iron uptake systems highlights the exceptional adaptability of these strains to iron-limited environments and suggests increased virulence potential.

In the analysis of *E. coli* isolates derived from domestic chickens, several toxin-associated genes were identified (Table 3). The *aslA* gene, encoding a protease–adhesin with hemagglutinin activity, was the most prevalent, found in 23 out of 87 isolates (36.5%). The *vat* gene—a vacuolating autotransporter toxin typically associated with APEC—was present in 3 isolates (4.8%), while *pic* and *stcE*, both encoding proteins with proteolytic and immune-modulating functions, were each detected in 1 isolate (1.6%).

Among the effector proteins, only *espY4*, a gene commonly linked to EPEC and EHEC strains, was detected, occurring in 10 isolates (15.9%). Other T3SS-related genes, including *espL*, *espR*, *espX*, and *espY*, were not detected in any of the isolates. The presence of *espY4* may suggest horizontal gene transfer or a mosaic virulence gene architecture.

### 2.2. Pathotypes

**Figure 1:**
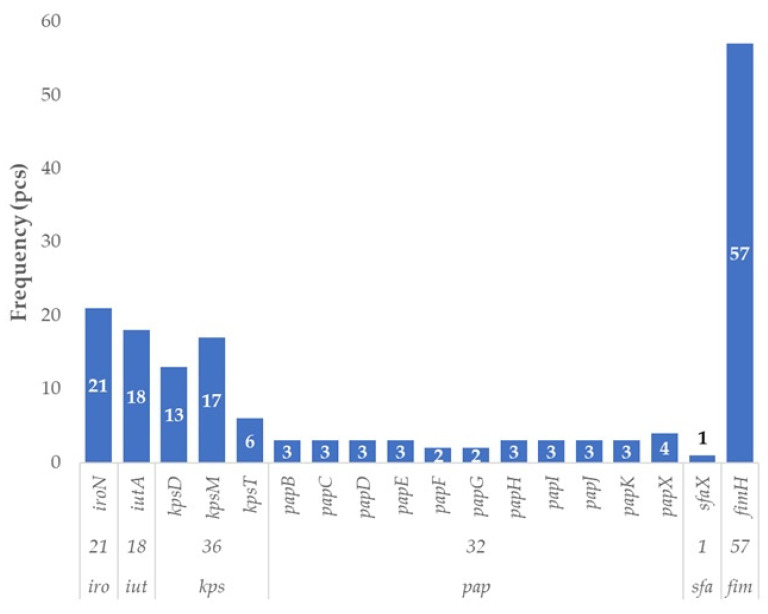
Prevalence of virulence factor genes characteristic of uropathogenic strains in *Escherichia coli* strains (UPEC) isolated from domestic chickens (*n* = 87). Among the 87 poultry-derived *E. coli* isolates, the *fimH* gene—encoding a type 1 fimbrial adhesin—was the most frequently detected UPEC-associated gene (57/87; 65.5%). Capsule synthesis genes (*kpsD*, *kpsM*, *kpsT*) were present in 36 isolates (41.4%), while iron acquisition genes such as *iroN* and iutA were found in 24.1% and 20.7% of strains, respectively. Although *pap* operon genes (e.g., *papC*, *papG*) and *sfaX* were less frequent, their presence indicates mosaic extraintestinal (ExPEC) virulence profiles. The overall distribution highlights the high prevalence of colonization and immune-evasion factors typical of ExPEC pathotypes in poultry strains, supporting their potential zoonotic relevance.

UPEC-associated virulence factors were identified in 58 out of 87 *E. coli* isolates originating from poultry, corresponding to a prevalence of 66.7%. This indicates that two-thirds of the strains harbored at least one gene typically linked to UPEC pathotypes (Figure 1). The most frequently detected gene was *fimH*, present in all but one of these strains (*n* = 57). Genes belonging to the *kps* family were found in 36 isolates, with *kpsM* being the most prevalent (*n* = 17), followed by *kpsD* (*n* = 13) and *kpsT* (*n* = 6). Members of the *pap* gene family were present in 32 strains, with *papX* being the most common (*n* = 4), while the remaining *pap* genes occurred in 2 to 3 strains each. The siderophore-associated genes *iroN* (*n* = 21) and *iutA* (*n* = 18), both linked to UPEC pathogenesis, were also frequently identified. In contrast, *sfaX* was detected in only a single isolate.

**Figure 2:**
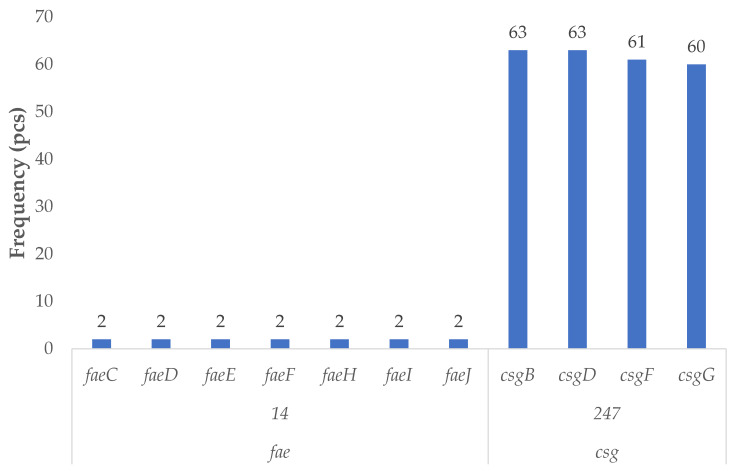
Prevalence of virulence factor genes characteristic of enterotoxigenic strains in *Escherichia coli* strains isolated from domestic chickens (*n* = 87). Curli fimbriae genes (*csgB*, *csgD*, *csgF*, *csgG*) were highly prevalent, occurring in more than 68% of isolates, highlighting strong colonization potential. Specifically, *csgB* and *csgD* were each detected in 63 strains (72.4%), followed closely by *csgF* (70.1%) and *csgG* (69.0%). These genes are involved in biofilm formation and epithelial adhesion. In contrast, the complete *fae* operon (F4/K88 fimbriae) was identified in only two isolates (2.3%), suggesting a rare but notable presence of classic enterotoxigenic (ETEC) adhesins. These findings suggest that while full enterotoxigenic profiles are uncommon, poultry *E. coli* may carry colonization-associated genes with pathogenic relevance and zoonotic implications.

Among the virulence factors associated with the ETEC pathotype (Figure 2), sixty-three poultry-derived isolates harbored genes belonging to this group. The curli fimbriae biosynthesis and regulatory genes *csgB* and *csgD* were identified in all sixty-three strains, while *csgF* and *csgG* were detected in sixty-one and sixty isolates, respectively. These genes are responsible for the assembly and secretion of surface fimbriae that contribute to bacterial adhesion.

In addition, genes involved in the biosynthesis of F4 (K88) fimbriae—a hallmark of classical ETEC strains—*faeC*, *faeD*, *faeE*, *faeF*, *faeH*, *faeI*, and *faeJ* were detected in both of the identified F4-positive isolates. The complete presence of this operon confirms their classification as F4-positive ETEC strains.

**Figure 3:**
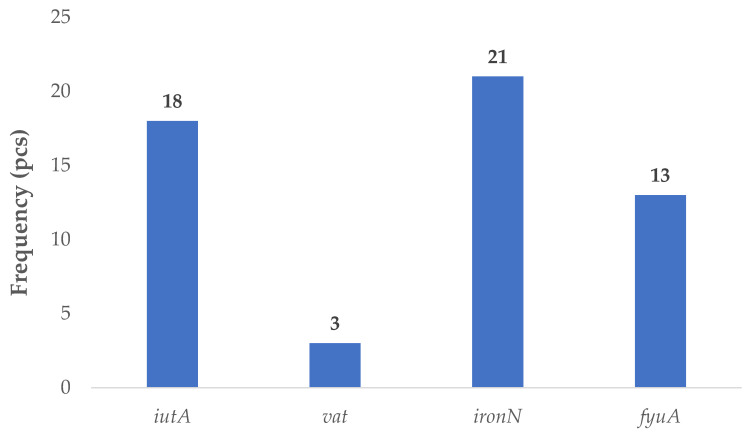
Prevalence of virulence factor genes characteristic of avian pathogenic (APEC) strains in *Escherichia coli* strains isolated from domestic chickens (*n* = 87). The salmochelin receptor gene *iroN* was the most frequently detected APEC-associated virulence gene, found in 21 isolates (24.1%), followed by *iutA* (20.7%), encoding the aerobactin receptor. The *fyuA* gene, which encodes the yersiniabactin receptor, was present in 13 strains (14.9%). The vacuolating autotransporter toxin gene vat appeared infrequently, identified in only 3 strains (3.4%). These findings suggest a heterogeneous distribution of APEC-associated virulence factors in the studied poultry population, with iron acquisition systems being particularly prominent. The data underscore the potential for extraintestinal disease development in avian hosts and raise concerns over zoonotic risk through shared virulence profiles with human extraintestinal (ExPEC) strains.

Virulence genes associated with the APEC pathotype were identified in thirty isolates. The *iutA* gene, encoding the receptor for the siderophore aerobactin, was present in eighteen strains. The *vat* gene, which encodes a vacuolating autotransporter toxin, was detected in three isolates. The *iroN* gene, part of the salmochelin iron acquisition system, was the most frequently identified, occurring in twenty-one strains. Additionally, the *fyuA* gene—encoding the receptor for the siderophore yersiniabactin—was found in thirteen isolates (Figure 3).

Among the poultry-derived *E. coli* strains analyzed, four isolates exhibited a gene combination characteristic of the neonatal NMEC pathotype. All four strains harbored the *ibeA* gene, a key invasion factor required for translocation across the blood–brain barrier. In addition, all four isolates carried the *fimH* gene, which is critical for epithelial adhesion. The capsule biosynthesis genes *kpsD*, *kpsM*, and *kpsT* were also identified and found more broadly across thirteen, seventeen, and six isolates, respectively.

**Figure 4:**
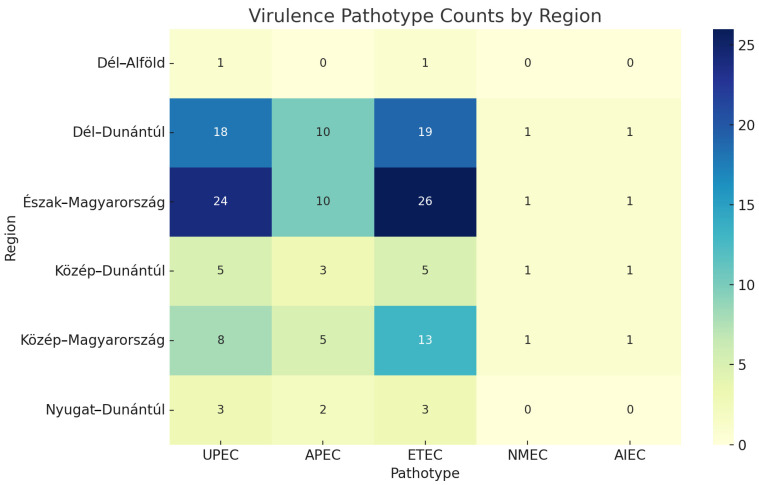
Distribution of five virulence-associated *Escherichia coli* pathotypes (UPEC—uropathogenic *Escherichia coli*, APEC—avian pathogenic *Escherichia coli*, ETEC—enterotoxigenic *Escherichia coli*, NMEC—neonatal meningitis-causing *Escherichia coli*, AIEC—adherent-invasive *Escherichia coli*) across poultry farms grouped by Hungary’s seven administrative regions. No statistically significant regional differences were observed (χ^2^ = 5.52, *p* = 0.999). Heatmap colors indicate the absolute number of isolates per category.

To assess potential spatial differences in the distribution of virulence-associated *E. coli* pathotypes, we compared their prevalence across Hungary’s seven administrative regions. The most common pathotypes were ETEC and UPEC, particularly in Észak-Magyarország and Dél-Dunántúl. A chi-squared test revealed no statistically significant association between region and pathotype distribution (χ^2^ = 5.52, df = 20, *p* = 0.999), suggesting a broadly uniform geographical spread of virulence traits among ESBL-producing strains (Figure 4).

## 3. Discussion

A total of 87 *E. coli* isolates were isolated from clinically healthy domestic chickens (*Gallus gallus domesticus*) reared in large-scale commercial broiler, layer, and breeder farms across Hungary. Following phenotypic pre-screening for multidrug resistance; results presented in a separate manuscript under review, we performed next-generation sequencing to elucidate the virulence gene repertoire of the isolates.

A substantial proportion of these poultry-derived *E. coli* isolates harbored colonization-related virulence factors typically associated with ExPEC *E. coli* pathotypes, particularly UPEC *E. coli*. Notably, the co-occurrence of genes belonging to the *fim* and *pap* operons, *ompA*, and the *E. coli* ECP operon suggests a high colonization potential within this collection. The *fimH* gene, in particular, merits attention due to its critical role in adhesion during urinary tract infections; previous studies have shown its presence in over 90% of UPEC isolates [30]. Members of the *pap* operon, especially *papG* and *papC*, are also instrumental in epithelial cell adherence and the development of ascending infections—an established role for P-fimbriae in ExPEC pathogenesis [31]. Interestingly, the presence of *fae* and *csg* gene families in these strains echoes traits seen in ETEC *E. coli*, reinforcing the notion of zoonotic potential. Previous studies have reported the presence of ETEC factors in poultry-derived *E. coli* strains; however, the complete fimbrial operon is rarely detected [32]. The detection of such elements raises public health concerns, particularly due to their frequent association with MGEs, which could facilitate interspecies gene transfer to human pathogens. This potential for genetic exchange may have implications for food safety and antimicrobial resistance dissemination, underscoring the importance of continuous genomic monitoring of virulence-rich poultry isolates [33].

Iron acquisition is a critical determinant of *E. coli* pathogenicity, enabling survival, colonization, and infection establishment within iron-restricted host environments—conditions shaped by nutritional immunity [34]. The detection of multiple siderophore systems in our isolates suggests robust adaptation to iron-limiting niches. The identification of both the *chu* and *shu* operons—heme utilization systems associated with ExPEC—points to functional redundancy in iron scavenging. Moreover, the diversity of siderophore systems identified, including enterobactin (*ent*/*fep*/*fes*), salmochelin (*iro*), yersiniabactin (*irp*/*ybt*), and aerobactin (*iuc*/*iut*), indicates high virulence potential and reinforces their zoonotic relevance [34]. Among these, *fyuA*, *iroN*, and *iutA* were frequently detected and are known markers of UPEC and APEC pathotypes, further suggesting that such strains may circulate within the poultry population. This extensive virulence arsenal in poultry-derived *E. coli* isolates supports concerns that such strains could act as potential reservoirs of virulence determinants relevant to public health, rather than directly confirming a zoonotic threat [35,36].

Comparable findings have been reported across Central and Eastern Europe, though the specific virulence gene combinations observed in this study appear distinct. For instance, Lithuanian and Polish poultry isolates showed ESBL-producing *E. coli* prevalence rates between 45% and 60%, often associated with APEC-type virulence markers rather than UPEC-related profiles [27]. In contrast, our Hungarian isolates—derived exclusively from healthy birds—displayed mixed virulence signatures that included ExPEC- and UPEC-characteristic genes, highlighting potential host adaptation and cross-pathotype evolution. Similar One Health surveillance work from Germany and Italy has also emphasized the increasing overlap between APEC and human ExPEC gene pools [28]. Collectively, these studies support a regional trend toward convergence of poultry and human-associated *E. coli* virulence determinants, suggesting ongoing zoonotic risk in European poultry production systems.

Bacterial toxins and effector proteins also play a pivotal role in modulating host cellular responses, immune evasion, and infection success. The detection of *aslA*, *pic*, and *stcE* genes in our study is consistent with ExPEC-specific pathogenic mechanisms. Notably, *aslA* has been implicated in blood–brain barrier translocation in both in vitro and in vivo models [37]. The Pic protease contributes to mucosal colonization through mucin layer degradation, a well-documented mechanism in enteroaggregative *E. coli* [38].

The presence of type III secretion system (T3SS) effectors—particularly those belonging to the *esp* gene family—is of special interest. These effectors are capable of modulating host signaling pathways to enhance bacterial persistence and replication. While the specific role of *espY4* in avian strains has not yet been characterized, its presence, previously documented in EPEC and EHEC strains, suggests novel effector activities with possible zoonotic implications [39,40], especially in strains exhibiting mosaic virulence gene content.

The detection of UPEC-associated virulence genes in poultry-derived *E. coli* isolates is particularly striking, as it highlights the avian population as a potential zoonotic reservoir. The near-universal detection of *fimH*—encoding the adhesin subunit of type 1 fimbriae—corroborates its well-established role in urinary tract epithelial adhesion, with a prevalence of up to 92.8% in clinical isolates [30]. Genes of the *kps* cluster, involved in capsule biosynthesis, provide protection against host immune responses and have been consistently observed in ExPEC strains [41]. The presence of *iroN* and *iutA* further supports the notion that these strains are well-equipped to survive in iron-depleted environments [42]. Although *pap* genes were less frequently identified—consistent with their predominance in human UPEC strains—their occurrence in avian isolates suggests zoonotic potential [43]. The detection of a single *sfaX*-positive isolate underscores the mosaic nature of ExPEC-associated virulence traits in poultry. Collectively, these findings point to the significant public health relevance of poultry *E. coli* as potential reservoirs of UPEC virulence determinants.

The detection of adhesion-associated virulence genes typical of the ETEC pathotype in poultry-derived isolates also raises potential zoonotic concerns [44]. The widespread presence of *csg* genes responsible for curli fimbriae biosynthesis (*csgB* and *csgD* were found in 100% of isolates) indicates robust colonization capabilities, particularly in facilitating adhesion to the intestinal epithelium [45]. Notably, two F4-positive (K88) strains were identified, both carrying the complete *fae* operon, responsible for the synthesis of K88 fimbriae—a hallmark of both human and animal ETEC strains [46]. While rare, this finding has significant implications: it suggests that poultry may serve as a reservoir not only for ExPEC but also for ETEC pathotypes. These strains possess considerable genetic potential for adhesion and colonization, posing risks to both animal and human hosts. Potential explanations include horizontal gene transfer (HGT) from pigs to poultry via MGEs such as plasmids or transposons, as well as cross-contamination in integrated animal husbandry systems.

The presence of APEC-associated virulence factors (*iroN*, *iutA*, *vat*, and *fyuA*) in 30 poultry-derived *E. coli* isolates further supports the notion that these strains can cause extraintestinal infections (e.g., colibacillosis) in avian hosts, raising additional zoonotic concerns [47]. The *iroN*, encoding a key component of the salmochelin siderophore system, was the most frequently detected gene and plays a central role in both APEC and UPEC pathogenesis, especially in invasive phenotypes [48]. The *iutA* gene, encoding the aerobactin receptor, was present in approximately 60% of isolates, consistent with previous reports identifying *iutA* as a major APEC marker [49]. Although *vat* was less common, its contribution to cellular damage underscores its relevance in disease pathology. The presence of *fyuA*, encoding a yersiniabactin receptor, reflects the diversity of iron acquisition systems—critical determinants of bacterial survival and virulence [50]. Altogether, these findings reinforce the view that poultry flocks may act as reservoirs for APEC strains, posing not only animal health risks but also a tangible zoonotic threat.

The detection of an NMEC-like gene profile (*ibeA*, *fimH*, *kpsD*/*M*/*T*) in four poultry isolates was unexpected, as these genes are typically associated with neonatal meningitis in humans [51]. The *ibeA* gene is particularly noteworthy due to its established role in promoting translocation across the blood–brain barrier via macrophage-mediated mechanisms [52]. The *fimH*, crucial for adhesion and colonization, was also present, along with the *kps* gene cluster (*kpsD*, *kpsM*, *kpsT*) required for the synthesis of the K1 capsule, a recognized NMEC virulence determinant [47]. Although NMEC strains are rarely documented in poultry, their detection here raises the possibility of zoonotic transmission. These findings underscore the need for comprehensive bacteriological surveillance and resistance monitoring in poultry production systems, aligned with the One Health framework.

Globally, reports from Asia indicate high prevalence and diverse virulence/resistance profiles in poultry-derived ESBL-producing *E. coli*. For example, a recent Malaysian study found 84.5% of chicken isolates positive for at least one ESBL gene [53], while in Taiwan, ESBL-producing poultry *E. coli* carried ExPEC-associated sequence types such as ST69 and ST617 [36]. These findings contrast with our results in Hungary, where, although the isolates were derived from healthy poultry, a high proportion harbored virulence factors typical of UPEC and APEC, rather than purely enteric pathotypes. This suggests regional variation in virulence gene distribution and highlights the importance of country-specific surveillance studies, particularly within the One Health framework.

In contrast, no key virulence genes associated with classical enteric pathotypes such as EHEC/STEC, EPEC, or DAEC were detected in any of the sequenced strains. Specifically, hallmark genes such as *stx1*, *stx2*, *eae*, *tir*, *bfp* (EHEC/EPEC), or *afa*/*dra* (DAEC) were entirely absent from the dataset. These results confirm that the analyzed poultry-derived *E. coli* isolates do not belong to enteric pathotypes but rather exhibit characteristics typical of extraintestinal strains [54].

Despite the numerical differences observed in pathotype counting across regions, no significant spatial clustering was detected. This uniform distribution might reflect the widespread movement of poultry stocks, shared hatchery sources, or similar selective pressures in farming practices across regions. The apparent dominance of ETEC and UPEC types aligns with their known prevalence in extraintestinal infections in both animals and humans.

In summary, a considerable proportion of the poultry-derived *E. coli* isolates in this study harbored virulence factors indicative of ExPEC potential—particularly genetic signatures characteristic of UPEC, APEC, and NMEC. In contrast, no markers associated with enteric pathotypes (EHEC/STEC, EPEC, DAEC) were detected, suggesting that current *E. coli* populations in poultry farming are predominantly extraintestinal in nature. These findings raise important public and animal health concerns, especially regarding zoonotic transmission and food chain safety, as supported by genomic studies highlighting overlaps between avian and human ExPEC strains, including the spread of high-risk clones like ST131 [55].

## 4. Materials and Methods

### 4.1. Sampling and Identification of Escherichia coli Strains

During the 2022–2023 period, biological samples were obtained from asymptomatic domestic chickens (*Gallus gallus domesticus*) housed on commercial poultry farms across Hungary. Farms were enrolled in the study on a voluntary basis and were selected to ensure geographic representativeness. A total of 23 sampling locations were visited, spanning all seven administrative regions of Hungary, with a minimum of three farms included per region to ensure adequate coverage. In most regions, exactly three farms were sampled, while in the Közép-Dunántúl region, five farms were included to enhance representativeness.

At each site, 15 tracheal and 15 cloacal swabs were collected per flock, based on practical considerations and to provide a representative sample of the microbial population. Sampling was performed as part of routine diagnostic monitoring by the attending veterinary officers. The swabs were collected using aluminum-shafted, non-charcoal Amies transport swabs (Biolab Zrt., Budapest, Hungary), following standard veterinary protocols.

The animals sampled were selected randomly from clinically healthy individuals within each flock, without prior knowledge of health status or antimicrobial exposure. No formal sample size calculation was performed, as the study was designed to be observational and exploratory in nature, aiming to detect the diversity of virulence factors present in poultry-derived *E. coli* strains on a national scale.

Samples were streaked onto ChromoBio^®^ Coliform agar (Biolab Zrt., Budapest, Hungary) to select presumptive *E. coli* colonies. Subsequent subculturing was performed on tryptone soya agar and incubated at 41 °C for 18–24 h. Identification of isolates was carried out by matrix-assisted laser desorption/ionization time-of-flight mass spectrometry (MALDI-TOF MS; Flextra-LAB Ltd., Budapest, Hungary) using Biotyper software v12.0 (Bruker Daltonics, Bremen, Germany) [56]. All confirmed strains were cryopreserved at −80 °C using Microbank™ storage systems (Pro-Lab Diagnostics, Richmond Hill, ON, Canada).

Phenotypic screening for antimicrobial resistance, including minimum inhibitory concentrations (MICs) testing and ESBL detection, was performed as part of the initial laboratory workflow. However, detailed results and interpretation of resistance profiles are beyond the scope of this study and are presented in a separate manuscript under review.

### 4.2. Next-Generation Sequencing

Genomic DNA was extracted from *E. coli* isolates using the Zymo Quick-DNA Fungal/Bacterial Miniprep Kit (Zymo Research, Irvine, CA, USA). Mechanical lysis was performed with a Qiagen TissueLyzer LT (Qiagen, Hilden, Germany). Extracted DNA was stored at −20 °C prior to sequencing. Whole-genome sequencing was carried out on the Illumina NextSeq 500 platform (Illumina, San Diego, CA, USA), generating paired-end reads using sequencing-by-synthesis chemistry.

DNA libraries were prepared using the Vazyme TruePrep DNA Library Prep Kit V2 (Vazyme, Nanjing, China) and Nextera XT Index Kits (Illumina, San Diego, CA, USA), followed by purification and quantification with Qubit dsDNA HS assays (Thermo Fisher Scientific, Waltham, MA, USA). For additional sequencing, libraries were adapted for the MGI DNBSEQ-G400RS platform using the MGIEasy Universal Library Conversion Kit and HotMPS High-Throughput chemistry (MGI Tech, Shenzhen, China). Circularization, DNB preparation, and quantification steps followed the manufacturer’s instructions.

### 4.3. Bioinformatic Analysis

Raw sequencing reads were evaluated using FastQC v0.11.9, Fastp v0.23.2-3, and Bloocoo v1.0.7 to assess base quality, adapter content, and sequencing depth. Low-quality reads were removed using TrimGalore v0.6.6.

De novo assembly was performed using MEGAHIT v1.2.9 and SPAdes v4.0.0 and merged with GAM-NGS v1.1b to improve assembly quality. The resulting contigs were assessed using QUAST v5.2 and BUSCO v5. Genome characteristics (e.g., k-mer distribution, genome size) were analyzed via GenomeScope v2.2.

Gene prediction was performed with Prodigal v2.6.3. Antimicrobial resistance genes (ARGs) were identified using RGI v5.1.0 and ABRicate against the CARD database, applying a ≥90% identity and coverage threshold.

MGEs were screened using MobileElementFinder v1.0.3. Putative plasmid-originated contigs and prophages were identified using PlasFlow v1.1 and VirSorter v2.2.2, respectively.

Taxonomic confirmation and completeness assessment were performed using CheckM v1.2.2 and Kraken v1.1.1. Chromosomal mutations were detected with ResFinder v4.1, and genome-wide SNPs were identified using Snippy v4.6.0. Serotype and virulence gene profiling were conducted using Ectyper v1.0 and VirulenceFinder v2.0, respectively.

Average Nucleotide Identity (ANI) was calculated with ANI v2.0, using *E. coli* SYNB8802 (RefSeq: GCF_020995495.1) as reference.

To explore potential geographical patterns in the distribution of virulence-associated *E. coli* pathotypes, each isolate was assigned to one of Hungary’s seven administrative regions, based on the location of the poultry farm where the sample originated. Pathotypes were classified as UPEC, APEC, ETEC, NMEC, or adherent-invasive *E. coli* (AIEC), according to the presence of specific virulence-associated genes identified through whole-genome sequencing.

The total number of isolates in each pathotype category was aggregated by region, and a contingency table was constructed to examine their spatial distribution. A chi-squared test of independence was applied to assess whether the distribution of pathotypes differed significantly among regions. Given the low counts observed for some categories (e.g., NMEC and AIEC), assumptions of the chi-squared test were verified, and Fisher’s exact test was considered as a more conservative alternative when appropriate.

All statistical analyses were carried out in R (version 4.3.1) using the base stats package and gmodels. Heatmaps visualizing the frequency of each pathotype by region were created using the ggplot2 v3.5.0 and reshape2 v1.4.4 packages. A *p*-value below 0.05 was considered statistically significant.

## 5. Conclusions

Comprehensive virulence profiling of the poultry-derived *E. coli* isolates revealed a clear predominance of genetic traits associated with ExPEC, particularly those linked to UPEC, APEC, and NMEC pathotypes. The complete absence of hallmark genes characteristic of enteric pathotypes (EHEC/STEC, EPEC, DAEC) further confirms that these isolates are primarily equipped to cause extraintestinal infections. This observation carries significant implications for zoonotic transmission and public health.

Our findings highlight that *E. coli* strains circulating in poultry flocks may represent a substantial risk not only to animal health but also to food chain integrity, especially when coupled with multidrug resistance phenotypes. Such dual pathogenic and antimicrobial resistance potential reinforces the urgency of implementing effective monitoring systems.

These findings provide actionable insights for One Health-oriented surveillance strategies by identifying specific virulence patterns circulating in poultry populations. The observed co-occurrence of ExPEC-, APEC-, and ETEC-associated genes in ESBL-producing *E. coli* underscores the necessity of genomic monitoring beyond resistance profiles alone. Such detailed virulome data could inform targeted vaccine development, particularly by prioritizing conserved colonization and iron acquisition factors like *fimH*, *iroN*, and *iutA*. In addition, the apparent regional similarities and differences, when compared to European and Asian datasets, highlight the need for harmonized surveillance protocols across borders. From a policy perspective, these insights support the rationale for reducing antimicrobial use in poultry farming to mitigate the zoonotic spillover of high-risk *E. coli* strains.

To mitigate these risks, sustained epidemiological surveillance, the routine application of high-throughput genomic screening, and the development of targeted intervention strategies are imperative—guided by the principles of the One Health approach. Future research should focus on characterizing potential transitions between pathotypes and identifying novel virulence gene combinations, with a view toward refining risk assessment and informing evidence-based control strategies.

## Figures and Tables

**Table 1:** Identified virulence factor genes of *Escherichia coli* pathotypes originating from domestic chickens, considering their colonization ability.

Class	VFG	Function	Pathotype
Colonization	*csgB*	Curli fimbriae structural subunit	ETEC
	*csgD*	Curli master regulator	ETEC
	*csgF*	Curli assembly	ETEC
	*csgG*	Curli secretion	ETEC
	*faeC*	K88 fimbriae biogenesis	ETEC
	*faeD*	K88 fimbriae assembly	ETEC
	*faeE*	K88 fimbriae assembly	ETEC
	*faeF*	K88 fimbriae assembly	ETEC
	*faeH*	K88 fimbriae structural subunit	ETEC
	*faeI*	K88 fimbriae assembly	ETEC
	*faeJ*	K88 fimbriae assembly	ETEC
	*fdeC*	Adhesin, epithelial cell binding	ExPEC
	*fimA*	Type 1 fimbriae, adhesion to epithelial cells	UPEC
	*fimB*	Phase variation regulator for fimbriae	UPEC
	*fimC*	Fimbrial chaperone	UPEC
	*fimD*	Fimbrial usher protein	UPEC
	*fimE*	Fimbrial regulator protein	UPEC
	*fimF*	Fimbrial component	UPEC
	*fimG*	Fimbrial minor subunit	UPEC
	*fimH*	Fimbrial adhesin, mannose-binding	UPEC
	*fimI*	Fimbrial subunit	UPEC
	*ompA*	Outer membrane protein, adhesion and immune evasion	ExPEC
	*papB*	Regulatory protein for P-fimbriae	UPEC
	*papC*	Fimbrial usher	UPEC
	*papD*	Fimbrial chaperone	UPEC
	*papE*	Fimbrial tip-associated subunit	UPEC
	*papF*	Fimbrial component	UPEC
	*papG*	Gal-binding adhesin	UPEC
	*papH*	Fimbrial anchoring subunit	UPEC
	*papI*	Regulatory protein	UPEC
	*papJ*	Fimbrial subunit	UPEC
	*papK*	Fimbrial subunit	UPEC
	*papX*	Regulatory protein	UPEC
	*yagV*/*ecpE*	*Escherichia coli* common pilus adhesion	UPEC
	*yagW*/*ecpD*	*Escherichia coli* common pilus usher protein	UPEC
	*yagX*/*ecpC*	*Escherichia coli* common pilus chaperone	UPEC
	*yagY*/*ecpB*	*Escherichia coli* common pilus subunit	UPEC
	*yagZ*/*ecpA*	*Escherichia coli* common pilus tip adhesin	UPEC
	*ykgK*/*ecpR*	*Escherichia coli* common pilus regulator	UPEC

VFG—virulence factor gene, UPEC—uropathogenic *Escherichia coli*; ExPEC—extraintestinal pathogenic *Escherichia coli*, ETEC—enterotoxigenic *Escherichia coli.*

**Table 2:** Identified virulence factor genes of *Escherichia coli* pathotypes originating from domestic chickens, in terms of fitness.

Class	VFG	Function	Pathotype
Fitness	*chuA*	Heme receptor for iron uptake	ExPEC
	*chuS*	Heme oxygenase	ExPEC
	*chuT*	Heme transport protein	ExPEC
	*chuU*	Heme ABC transporter component	ExPEC
	*chuV*	Heme ABC transporter ATPase	ExPEC
	*chuW*	Putative heme utilization protein	ExPEC
	*chuX*	Heme-binding protein	ExPEC
	*chuY*	Putative heme degradation	ExPEC
	*entA*	Enterobactin biosynthesis (siderophore)	ExPEC
	*entB*	Enterobactin biosynthesis (siderophore)	ExPEC
	*entC*	Enterobactin biosynthesis (siderophore)	ExPEC
	*entD*	Enterobactin biosynthesis (siderophore)	ExPEC
	*entE*	Enterobactin biosynthesis (siderophore)	ExPEC
	*entF*	Enterobactin biosynthesis (siderophore)	ExPEC
	*entS*	Enterobactin exporter	ExPEC
	*fepA*	Enterobactin receptor	ExPEC
	*fepB*	Enterobactin-binding periplasmic protein	ExPEC
	*fepC*	ABC transporter ATPase	ExPEC
	*fepD*	ABC transporter permease	ExPEC
	*fepG*	ABC transporter permease	ExPEC
	*fes*	Enterobactin esterase	ExPEC
	*fyuA*	Yersiniabactin receptor	APEC
	*iroB*	Salmochelin glycosyltransferase	ExPEC
	*iroC*	Salmochelin exporter	ExPEC
	*iroD*	Salmochelin esterase	ExPEC
	*iroE*	Salmochelin hydrolysis	ExPEC
	*iroN*	Salmochelin receptor	APEC, UPEC
	*irp1*	Yersiniabactin biosynthesis	ExPEC
	*irp2*	Yersiniabactin biosynthesis	ExPEC
	*iucA*	Aerobactin biosynthesis	ExPEC
	*iucB*	Aerobactin biosynthesis	ExPEC
	*iucC*	Aerobactin biosynthesis	ExPEC
	*iucD*	Aerobactin biosynthesis	ExPEC
	*iutA*	Aerobactin receptor	APEC
	*shuA*	Heme receptor (homologous to chuA)	ExPEC
	*shuS*	Heme utilization protein	ExPEC
	*shuT*	Heme ABC transporter periplasmic component	ExPEC
	*shuX*	Putative heme-binding protein	ExPEC
	*shuY*	Putative heme utilization	ExPEC
	*ybtA*	Yersiniabactin regulator	ExPEC
	*ybtE*	Yersiniabactin biosynthesis	ExPEC
	*ybtP*	Yersiniabactin ABC transporter	ExPEC
	*ybtQ*	Yersiniabactin ABC transporter	ExPEC
	*ybtS*	Yersiniabactin biosynthesis	ExPEC
	*ybtT*	Yersiniabactin biosynthesis	ExPEC
	*ybtU*	Yersiniabactin biosynthesis	ExPEC
	*ybtX*	Yersiniabactin efflux	ExPEC

VFG—virulence factor gene, UPEC—uropathogenic *Escherichia coli*, ExPEC—extraintestinal pathogenic *Escherichia coli*, APEC—avian pathogenic *Escherichia coli.*

**Table 3:** Identified virulence factor genes of *Escherichia coli* pathotypes originating from domestic chickens, in light of toxins and effectors.

Class	VFG	Function	Pathotype
Toxins	*aslA*	Putative autotransporter, hemagglutinin	ExPEC
	*pic*	Serine protease autotransporter	ExPEC
	*stcE*	Metalloprotease, modifies host mucins	ExPEC
	*vat*	Vacuolating autotransporter toxin	APEC
Effectors	*espL1*	Type III secretion effector	ExPEC
	*espL4*	Type III secretion effector	ExPEC
	*espR1*	Type III secretion effector	ExPEC
	*espR3*	Type III secretion effector	ExPEC
	*espR4*	Type III secretion effector	ExPEC
	*espX1*	Type III secretion effector	ExPEC
	*espX2*	Type III secretion effector	ExPEC
	*espX4*	Type III secretion effector	ExPEC
	*espX5*	Type III secretion effector	ExPEC
	*espX6*	Type III secretion effector	ExPEC
	*espY1*	Type III secretion effector	ExPEC
	*espY2*	Type III secretion effector	ExPEC
	*espY3*	Type III secretion effector	ExPEC
	*espY4*	Type III secretion effector	ExPEC
	*espY4*	Type III secretion effector	EPEC, EHEC

VFG—virulence factor gene, ExPEC—extraintestinal pathogenic *Escherichia coli*, APEC—avian pathogenic *Escherichia coli*, EPEC—enteropathogenic *Escherichia coli*, EHEC—enterohemorrhagic *Escherichia coli*.

## Data Availability

The datasets used and/or analyzed during the current study are available from the corresponding author on reasonable request. The sequencing files are available at the https://www.ncbi.nlm.nih.gov/bioproject/PRJNA1332072, accessed on 20 September 2025.

## Supplementary Materials

The following supporting information can be downloaded at https://www.mdpi.com/article/10.3390/antibiotics14111083/s1, Additional data (chickens).
